# Athletes’ basic psychological need satisfaction and autonomous motivation: differences between individual vs. team sports

**DOI:** 10.3389/fspor.2025.1592356

**Published:** 2025-11-11

**Authors:** Nico W. Van Yperen

**Affiliations:** Department of Psychology, University of Groningen, Groningen, Netherlands

**Keywords:** Self-Determination, motive, autonomy, competence, relatedness, support

## Abstract

Self-Determination Theory (SDT) posits that the fulfillment of three basic psychological needs—autonomy, competence, and relatedness—is essential for fostering autonomous motivation, well-being, and optimal functioning. The present research aimed to extend current understanding of how sport modality (individual vs. team) relates to these sources of autonomous motivation in athletes. Data were collected across two studies: Study 1 included tennis and volleyball players (*n* = 78), while Study 2 involved a larger and more diverse sample of individual and team sport athletes (*n* = 1,137). Analyses of covariance revealed that individual sport athletes reported higher autonomy satisfaction. In contrast, team sport athletes reported higher relatedness satisfaction and, in Study 2 only, higher competence satisfaction. As anticipated, differences emerged in the sources of autonomous motivation rather than in the overall strength of autonomous motivation itself. These findings provide valuable insights into athletes’ psychological need satisfaction profiles and offer a practical framework for implementing need-supportive coaching practices tailored to sport type.

## Introduction

In the sport domain, autonomous forms of motivation have been found to be key factors in the explanation of critical outcome variables such as vitality and active living (1, 2), high quality performance and growth [e.g., (3–5)], and persistence [e.g., (6–8)]. In Self-Determination theory (SDT), autonomous motivation is defined as engagement in an activity because it is perceived as congruent with intrinsic goals and stems from the self (9). SDT states that the source for the development and maintenance of autonomous motivation is the fulfillment of the basic psychological needs for autonomy, competence, and relatedness. Fulfillment of the need for *autonomy* refers to the experience of volition and psychological freedom. Individuals’ need for *competence* is satisfied when experiencing mastery and effectiveness in one's pursuits. The experience of *relatedness* satisfaction is defined by the sense of connection with other human beings and mutual trust and concern (10, 11).

In SDT, fulfillment of these three psychological needs is considered as universal nutriment necessary for autonomous motivation, and accordingly, well-being, optimal functioning, and a healthy development across demographics, psychological characteristics, and cultural contexts (10, 12). This universality claim, however, does not contradict the idea that there exists considerable environmental variation in the extent to which the different basic psychological needs are fulfilled [e.g., (13). In the words of Ryan and Deci (9), p.88]: “*…. groups differ—for example, with some espousing the primacy of the group over the individual and others espousing the primacy of the individual over the group.*” The assertion that basic needs are universal includes the recognition that the satisfaction of basic needs can be accomplished in varied ways in different social contexts through different cultural forms [cf., (12, 14)]. Hence, the pattern of athletes’ psychological need satisfaction may be different in individual vs. team sports.

To date, differences between individual and team sport environments in how conductive they are to the fulfillment of basic human psychological needs, is a neglected issue in the psychological literature. To fill this gap, in the present study, we draw on this major contextual dichotomy in sports by examining the link between sport modality (individual vs. team) and the sources of autonomous sport motivation. Specifically, we will argue and demonstrate that relative to individual sports, athletes in team sports are lower in autonomy satisfaction, and higher in relatedness and competence satisfaction, which will be discussed in more detail next.

## Psychological need satisfaction in individual vs. team sports

In individual sports, the personal responsibility for the outcome is higher relative to team sports [e.g., (15)]. In individual sports, the athlete is solely responsible for the result whereas in team sports, the final result relies on the whole team. Hence, more than team sports athletes, individual sports athletes pursue independently their own individual goals without being responsible to a team. This likely results in higher autonomy satisfaction among individual sports athletes than athletes from team sports [e.g., (16)].

As demonstrated by Nixdorf et al. (17), however, a downside of autonomy may be a pattern of negative internal attributions after failure, which is indeed more common in individual sports than in team sports [e.g., (18)]. In team sports, athletes are collectively responsible for their team's failures and successes. For them, explaining failures and successes is more ambiguous than for individual sports athletes who are solely responsible for their results. More ambiguity provides more degrees of freedom for self-serving attribution biases and self-enhancement [e.g., (19)]. That is, after team defeats, team sports athletes have more opportunities to maintain positive beliefs about themselves by attributing failures to their co-responsible teammates. Indeed, meta-analyses revealed that team sports athletes tend to claim more personal responsibility for team success and less personal responsibility for team failure (20, 21). Furthermore, relative to team sports athletes, the prevalence of negative emotions, including (competitive) anxiety, depression, guilt, and shame is higher among individual sports athletes [e.g., (17, 22–26)]. Therefore, relative to individual sports athletes, competence satisfaction can be expected to be more pronounced in team sports athletes.

In addition, team sports athletes’ psychological need satisfaction may be derived from compliance, trust, connectedness, and sociotropy that are inherent nutriments in team sports. Team sports athletes work together towards a common goal, which often enhances their communication skills, sense of positive mutual interdependency, mutual care and concern for each other, and reinforces feelings of collectivity and team cohesion [e.g., (16, 27–29)]. These team dynamics will increase team sport athletes’ need for relatedness satisfaction. To illustrate, in situations that thwart their need for relatedness (e.g., pandemic-induced lockdowns characterized by social isolation and lack of social team activity), team sports athletes reported higher levels of mental health problems than individual sports athletes (30).

In sum, in the present research, we tested the hypotheses that relative to individual sports athletes, team sports athletes are lower in *autonomy* satisfaction (*Hypothesis 1*), higher in *competence* satisfaction (*Hypothesis 2*), and higher in *relatedness* satisfaction (*Hypothesis 3*). Note that there is no reason to assume that the positive associations between need satisfaction and autonomous motivation—or the strength of autonomous sport motivation—differ between individual and team sports. Differences are expected only in the levels of the three types of psychological need satisfaction that foster autonomous motivation.

In Study 1, we tested the three hypotheses in a sample of tennis players vs. volleyball players (*n* = 78). Tennis is predominantly an individual sport whereas volleyball is an interactive team sport. Both sports, however, are similar in terms of *net play* [i.e., the player(s) on opposite sides try to keep the ball in play and score points by hitting it over the net], *serving* (i.e., a well-executed serve can put pressure on the opponent and set the tone for the point), and *scoring* [i.e., player(s) earn points when the opposing side fails to return the ball properly]. To strengthen both the statistical power and the external validity, Study 2 relied on a much larger sample (*n* = 1,137) that included athletes from a variety of individual and team sports.

## Method Study 1

### Power analysis

We conducted two *a priori* power analyses with the statistical power analysis program G*Power 3.1 (31). In the first analysis, the input parameters were a linear multiple regression with two tails, an alternative hypothesis assuming a population squared multiple correlation of .30, a null hypothesis assuming a population squared multiple correlation of 0, an alpha error probability of .05, a statistical power of .95, and a total of seven predictors. This resulted in a required sample size of 71. However, our main analyses are analyses of covariance. Therefore, in the second analysis, the input parameters were an *F* test for an analysis of covariance with Cohen's *f* effect size of .50 (32), an alpha error probability of .05, a statistical power of .95, one numerator degree of freedom, two groups, and four covariates. This resulted in a required sample size of 55.

### Participants

The final sample consisted of 78 athletes (53.8% women) playing either tennis (43.6%) or volleyball. The mean age was 27.91 years (SD =13.43), ranging from 16 to 58. Current level of play varied from recreational/regional level (84.6%), national sub-top level (10.3%), to national top level (5.1%). In the 12 months before filling out the questionnaire, the number of training hours per week (at their club, selection and/or for themselves) ranged from 0 to 15 (*M* = 3.52, SD = 2.70).

### Procedure

After having obtained approval from the university's ethical committee, tennis players and volleyball players were recruited using a convenience sample method. Specifically, in the context of their research project, undergraduates approached athletes from their personal network online (e.g., via Whatsapp) and posted announcement on social media (e.g., Facebook, Instagram). Participation was voluntary and participants received no incentives. After following the hyperlink to the Qualtrics questionnaire on “Sport Motivation”, participants were informed about the nature and the procedure of the study. About 24% was excluded from the analyses because they had not provided explicit informed consent prior to filling out the survey, had not indicated to play either tennis or volleyball, were younger than 16 years of age (i.e., those who needed to have formal approval from their parents), did not have a complete dataset, or had indicated at the end of the survey that they (1) had not answered all the questions honestly, or (2) had not read and answered all the questions carefully [e.g., (33, 34)].

### Measures

For all multi-item measures, scale scores were obtained by averaging the scores on the individual items.

**Psychological need satisfaction.** The degree to which athletes experienced satisfaction of the three psychological needs was assessed using nine items derived from three previously validated questionnaires (35–37). Responses were scored on a seven-point Likert scale which ranged from (1) *not at all*, to (7) *to an extremely high extent*. Note that in the used items, participants read either “In tennis” or “In volleyball” rather than “In my sport”. This was obviously contingent on the sport they played, what they had indicated at the beginning of the digitalized questionnaire by answering the question: “What is your main sport?”

The three items used for assessing *autonomy* satisfaction were: (1) In my sport, I have a say in things that are important to me; (2) In my sport, I can decide for myself what is good for me as an athlete; (3) In my sport, I feel free to make my own choices. Cronbach's alpha was .70.

The three-item scale for *competence* satisfaction was: (1) In my sport, I feel I have the knowledge and skills to execute my tasks well; (2) I feel competent in my sport; (3) Overall (technically, physically, mentally), I am good at my sport. Cronbach's alpha was .88.

Finally, *relatedness* satisfaction was assessed using the following three items: (1) In my sport, I can go to others when I am struggling with something; (2) In my sport, I have real friends; (3) In my sport, I feel part of a team or group. Cronbach's alpha was .84.

**Autonomous sport motivation** was assessed with the revised Sport Motivation Scale (SMS-II) developed and validated by Pelletier et al. (2). The concept of autonomous motivation includes intrinsic regulation and internalized forms of extrinsic motivation [i.e., identified and integrated regulation; (10)]. *Intrinsic regulation* is actually another label for intrinsic motivation, where the motivation for acting derives from satisfactions found in the behavior itself (2). Also both forms of extrinsic motivation are accompanied by high levels of perceived autonomy and a sense of personal commitment and engagement. Specifically, in *identified regulation*, one's behavior is experienced as personally important and worthwhile whereas *integrated regulation* occurs when the behavior is not only seen as valued, but also as congruent with the individual's other life goals, objectives, and needs. In research, typically, one index for autonomous motivation is used by averaging the sub-scales of autonomous motivation [e.g., (38–42)]. In the present study, Cronbach's alpha of the autonomous sport motivation composite was .88.

### Results Study 1

All analyses were conducted using SPSS Statistics software, version 29. Table 1 shows that age was negatively associated with training hours, relatedness satisfaction, and competence satisfaction. Obviously, athletes competing at a higher level reported more weekly training hours. Simple *t*-tests further revealed one sex difference (*p* < .05): relative to male athletes (*M* = 4.06, SD = 1.35), female athletes (*M* = 5.03, SD = 1.00) were higher in relatedness satisfaction, *t*(61.37) = 3.55, *p* < .001.

**Table 1:** Means, standard deviations, and Pearson's correlations in Study 1 (below the diagonal) and Study 2 (above the diagonal).

Variable	*M*_Study 1_	*SD*_Study 1_	*M*_Study 2_	*SD*_Study 2_	1	2	3	4	5	6	7
1. Age	27.91	13.43	29.82	14.12	–	−0.03	−.18	−.03	−.28	−.24	−.05
2. Level of play	1.21	.52	4.83	.65	.26	–	.30	.08	−.10	.04	.05
3. Training hours per week	3.52	2.70	4.30	3.73	−.30	.59	–	.12	.07	.18	.18
4. Autonomy satisfaction	4.82	.91	4.79	.89	−.18	−.16	.11	–	.11	.31	.22
5. Relatedness satisfaction	4.57	1.26	5.10	1.26	−.35	.19	.23	.16	–	.33	.27
6. Competence satisfaction	4.57	.94	4.73	.90	−.33	.29	.48	.31	.49	–	.21
7. Autonomous motivation	4.87	.97	5.09	.90	−.15	.11	.28	.47	.42	.30	–

In Study 1 (*n* = 78), correlations higher than .24 and .29 (in absolute values) are significant at the *p* = .05 and *p* = .01 level, respectively.

In Study 2 (*n* = 1,137), correlations higher than .08 (in absolute values) are significant at the *p* = .01 level.

In line with SDT, Table 1 shows that all three measures of basic need satisfaction were positively correlated with autonomous motivation. To check the assumption that sport modality does not moderate these associations, we ran three ordinary least squares (OLS) linear regression analyses, one for each need. In all models, autonomous motivation served as the dependent variable. Predictor variables included the centered score of the respective need satisfaction, sport modality (tennis vs. volleyball), and their interaction term. In addition, sex, age, level of play, and weekly training hours were entered as covariates. While the main effects of autonomy and relatedness satisfaction were highly significant (*p*s < .001), and competence satisfaction reached significance (*p* = .04; see also Table 1), none of the interaction terms were significant (*p*s > .21). Thus, the positive associations between need satisfaction and autonomous motivation (see also Table 1) did not differ between tennis and volleyball.

*Hypothesis 1* stated that relative to individual sports athletes, team sports athletes are lower in autonomy satisfaction. To test this hypothesis, we conducted a univariate analysis of covariance (ANCOVA) with sport (tennis vs. volleyball) as the independent variable, autonomy satisfaction as the dependent variable, and sex, age, level of play, and training hours per week as covariates. As shown in Table 2, this analysis revealed a significant effect of Sport, indicating that 15% (*η*^2^ = .15) of the variance of autonomy satisfaction was attributable to the sport modality. That is, relative to volleyball players, tennis players were higher in autonomy satisfaction, which provided empirical support for *Hypothesis 1*.

**Table 2:** Mean differences between team sport (volleyball) and individual sport (tennis) athletes (Study 1).

	Volleyball (*n* = 34)		Tennis (*n* = 44)			
Variable	*M*	SE	*M*	SE	*F*(1, 72)	*η* ^2^
Autonomy satisfaction	4.38 [4.08–4.69]	.15	5.16 [4.90–5.42]	.13	12.83***	.15
Competence satisfaction	4.71 [4.40–5.02]	.16	4.46 [4.19–4.73]	.14	1.23	.02
Relatedness satisfaction	4.94 [4.52–5.36]	.21	4.29 [3.93–4.65]	.18	4.77*	.06
Autonomous motivation	4.65 [4.30–5.01]	.18	5.03 [4.72–5.34]	.15	2.19	.03

Presented are the means, including the 95% confidence interval in square brackets, adjusted for sex, age, level of play, and training hours per week. Effect sizes generated by AN(C)OVAs (*η*^2^) can be interpreted as follows [(32), pp. 283–288]: around .01 = “small,” around .06 = “moderate”, and around .14 = “large”.

* *p* < .05

** *p* < .01

*** *p* < .001.

To test the other two hypotheses, we reran the same analysis twice, replacing autonomy satisfaction with competence satisfaction and relatedness satisfaction, respectively. Table 2 shows that in Study 1, no empirical support was found for *Hypothesis 2*. Although the (adjusted) means were in the expected direction, volleyball players were not significantly higher in *competence* satisfaction than tennis players. In line with *Hypothesis 3*, however, volleyball players were higher in *relatedness* satisfaction than tennis players. Finally, as expected, no differences between sports in autonomous motivation were observed (see Table 2).

### Study 2

In Study 1, the findings were largely in line with the expectations, but the sample was limited and individual and team sports athletes represented only one sport: tennis and volleyball, respectively. Furthermore, studies with small sample sizes may suffer from false-positive results and inflated effect sizes (43). Hence, in Study 2, data were collected from a large sample of athletes (*n* = 1,137) representing a wide range of individual and team sports. This broader sample provides greater diversity across sport types and makes it possible to examine whether the findings extend beyond tennis and volleyball.

## Method Study 2

### Participants and procedure

The same procedure as in Study 1 was followed. However, to examine whether the findings extend beyond tennis and volleyball, in Study 2, athletes from various individual and team sports, except tennis and volleyball, were recruited. For the same reasons as in Study 1, we excluded about 27% of the participants. In Study 2, participants were included if they had indicated to play either an individual or team sport, except tennis or volleyball. The final sample comprised 363 athletes (31.9%) from various individual sports (e.g., gymnastics, martial arts, track, badminton, fitness, dams). The 774 team sport athletes players were involved in sports such as basketball, field hockey, korfball, and soccer.

Age of the 1,137 athletes (56.3% women) ranged from 16 to 68 (*M* = 29.82, SD = 14.12). Current level of play varied from recreational/regional level (92.4%), national sub-top level (1%), national top level (4.9%), to international (top) level (1.9%). In the 12 months before filling out the questionnaire, the number of training hours per week (at their club, selection and/or for themselves) ranged from 0 to 40 (*M* = 4.30, SD = 3.73).

In Study 2, we relied on the same measures as in Study 1. Similar Cronbach's alpha's were observed: .71 (autonomy satisfaction), .87 (competence satisfaction), .84 (relatedness satisfaction), and .86 (autonomous motivation).

### Results Study 2

Consistent with Study 1, Table 1 shows that age was negatively associated with training hours, relatedness satisfaction, and competence satisfaction, and athletes at higher competitive levels engaged in more weekly training hours. Furthermore, in Study 2, a simple t-test again indicated that relative to male athletes (*M* = 4.89, SD = 1.25), female athletes (*M* = 5.25, SD = 1.24) were higher in relatedness satisfaction, *t*(1,135) = 4.84, *p* < .001.

In contrast to Study 1, however, male athletes were older [*M*_males_ = 34.88, SD = 16.07 vs. *M*_females_ = 25.89, SD = 10.89, *t*(830.89) = 10.71, *p* < .001], trained more hours per week [*M*_males_ = 4.68, SD = 4.04 vs. *M*_females_ = 4.01, SD = 3.43, *t*(970.78) = 2.98, *p* < .01], and were higher in autonomy satisfaction [*M*_males_ = 4.94, SD = .87 vs. *M*_females_ = 4.67, SD = .89, *t*(1,135) = 5.09, *p* < .001].

Table 1 further shows that also in Study 2, basic needs satisfaction was positively correlated with autonomous motivation, which is in line with SDT. As in Study 1, we next performed the same three follow-up regression analyses to check the assumption that sport modality does not moderate these associations. Also in Study 2, the main effects of need satisfaction (*p*s < .001) were significant, and the interaction terms were not significant (*p*s > .26). Thus, also in Study 2, we found that the significant and positive links between psychological need satisfaction and autonomous motivation (see Table 1) were not different in individual and team sports.

To test the three hypotheses, we conducted the same analyses as in Study 1. Specifically, we performed three univariate analyses of covariance (ANCOVAs) with sport (individual vs. team) as the independent variable, sex, age, level of play, and training hours per week as covariates, and autonomy satisfaction (*Hypothesis 1*), competence satisfaction (*Hypothesis 2*), and relatedness satisfaction (*Hypothesis 3*) as the dependent variable, respectively. As in Study 1, individual sports athletes were higher in autonomy satisfaction (*Hypothesis 1*) whereas team sports athletes were higher in relatedness satisfaction (*Hypothesis 3*). Furthermore, Table 3 shows that in Study 2, we found the hypothesized (*Hypothesis 2*) difference in competence satisfaction as well. That is, team sports athletes were higher in competence satisfaction than individual sports athletes. In Study 2, 6% (*η*^2^ = .06) of the variance of competence satisfaction was attributable to sport modality. Thus, in Study 2, empirical support was found not only for *Hypotheses 1 and 3* (as in Study 1), but also for *Hypothesis 2*. Table 3 further shows that also in Study 2, the strength of autonomous motivation did not differ between team and individual sports athletes.

**Table 3:** Mean differences between team sport and individual sport athletes (Study 2).

	Team sports (*n* = 774)		Individual sports (*n* = 363)			
Variable	*M*	SE	*M*	SE	*F*(1, 72)	*η* ^2^
Autonomy satisfaction	4.67 [4.60–4.73]	.03	5.05 [4.95–5.15]	.05	35.16***	.03
Competence satisfaction	4.89 [4.83–4.95]	.03	4.38 [4.29–4.48]	.05	66.85***	.06
Relatedness satisfaction	5.48 [5.40–5.57]	.04	4.27 [4.14–4.39]	.07	219.53***	.16
Autonomous motivation	5.10 [5.03–5.16]	.03	5.08 [4.98–5.19]	.05	.04	.00

Presented are the means, including the 95% confidence interval in square brackets, adjusted for sex, age, level of play, and training hours per week. Effect sizes generated by AN(C)OVAs (*η*^2^) can be interpreted as follows [(32), pp. 283–288]: around .01 = “small,” around .06 = “moderate,” and around .14 = “large”.

* *p* < .05

** *p* < .01

*** *p* < .001.

## Discussion

Self-Determination theory (SDT) considers the fulfillment of the three basic psychological needs for autonomy, competence, and relatedness as universal nutriment necessary for autonomous motivation, and accordingly, well-being and optimal functioning. The fulfillment of the basic psychological needs, however, can be accomplished in varied ways in different social contexts through different cultural forms [e.g., (9, 12, 14)]. Drawing on a major contextual dichotomy in sports (i.e., individual vs. team sports), the current findings suggest that relative to individual sports, athletes in team sports are lower in autonomy satisfaction, and higher in relatedness and competence satisfaction. And only these sources, not autonomous motivation itself, appeared to differ between individual and team sports athletes.

The current findings also show that in both individual and team sports, higher levels of basic psychological need satisfaction is accompanied with higher levels of autonomous motivation. As expected, sport modality did not moderate these associations. The difference between individual and team sports is that these positive correlations occur at different levels of need satisfaction within each type of sport. That is, positive links between autonomy satisfaction and autonomous motivation exist at higher levels of autonomy satisfaction among individual sport athletes, and among team sports athletes, at higher levels of relatedness and competence satisfaction. Phrased differently, individual sports athletes draw their autonomous motivation from higher levels of need for autonomy satisfaction whereas team sports athletes derive similar levels of autonomous motivation from higher levels of relatedness and competence satisfaction. Important to note, however, is that across both types of sports, the higher the satisfaction of each psychological need, the higher athletes’ autonomous motivation (10, 11). In future studies, it may be tested whether both types of sports are similar in terms of (subsequent) levels of well-being, optimal functioning, and a healthy development as well [e.g., (1, 10, 44)].

An important question is why individual and team sport athletes differ in their levels of psychological need satisfaction. Compared to team sport athletes, individual sport athletes typically report higher autonomy satisfaction, likely because they are more often provided with autonomy-supportive environments by their coaches [e.g., (45)]. One explanation is that athletes in individual sports bear sole responsibility for performance outcomes, which may foster a greater sense of personal agency and ownership (15). In contrast, in team sports, performance outcomes depend on the collective efforts of the team. This shared responsibility promotes a sense of interdependence, reinforcing feelings of collectivity and team cohesion, which may increase relatedness satisfaction. At the same time, team dynamics can introduce ambiguity regarding individual contributions to success or failure. This ambiguity may create greater opportunity for self-serving attribution biases and self-enhancement processes [e.g., (19)], which could explain why team sport athletes tend to report higher competence satisfaction than their individual sport counterparts.

Note that the universal hypothesis in SDT implies that individual differences in need strength are considered as not existing and irrelevant [e.g., (46)]. However, another theory of psychological needs, Motive Disposition Theory [MDT; (47)], conceptualized needs as early acquired and relatively stable motive dispositions that vary from person to person (48, 49). From this latter perspective, athletes high in need for autonomy may feel more attracted to individual sports whereas individuals high in needs for relatedness and competence likely prefer to get involved in team sports. These individuals, in turn, likely receive the support within their “fitting” sport environment that is congruent with their needs. Indeed, MDT's individual difference perspective and SDT's environmental view are not mutually exclusive. MDT's psychological *need strength* predicts individuals’ preference for individual or team sports whereas SDT predicts psychological *need satisfaction* on the basis of sport modality.

### Practical implications

This research provides a valuable roadmap for implementing need-based coaching and athlete support strategies [e.g., (1, 50–53)]. Recognizing that different sport modalities (individual vs. team) tend to fulfill distinct psychological needs enables practitioners to more effectively foster autonomous motivation in athletes. Accordingly, support systems can be strategically tailored to align with sport type and the psychological needs most salient within each modality.

When an athlete shows signs of low autonomous motivation, coaches and sport psychologists can examine which basic psychological need—autonomy, competence, or relatedness—may be insufficiently fulfilled, considering the nature of the sport. For example, in individual sports, coaches can support autonomy by involving athletes in planning, providing meaningful feedback, and encouraging self-reflection to promote ownership of their training and performance goals. In contrast, in team sports, it may be more effective to enhance relatedness satisfaction by emphasizing interpersonal connectedness, mutual support, and the pursuit of shared goals. Additionally, recognizing both individual contributions and collective achievements can further strengthen athletes’ feelings of competence and relatedness.

Moreover, coaches may intentionally design training environments that *compensate* for needs that may be less organically met within a given sport modality. For instance, because individual sport athletes may struggle with a sense of relatedness due to the inherently solo nature of their disciplines, coaches might build peer support networks, create mentorship opportunities, or structure shared training and recovery routines [e.g., (54, 55)]. On the other hand, since autonomy may be less naturally supported in team sports, coaches may promote role flexibility, decision-making opportunities, and personal accountability within the team structure.

### Strengths and limitations

The present research has at least three main strengths. First, we add to the extant literature on SDT by showing in two different studies that individual sport athletes are higher in autonomy satisfaction whereas team sport athletes are higher in relatedness satisfaction, and, only in Study 2, in competence satisfaction. Second, our findings suggest that individual and team sports considerably differ in the extent to which the different basic psychological needs are fulfilled and that autonomous motivation can be strengthened by following these different paths. A third strength of the present research is that similar patterns were observed across two different samples: one consisting of tennis and volleyball players, and another larger and more diverse sample including athletes from a variety of other individual and team sports. This provides empirical evidence that the findings extend beyond tennis and volleyball.

Balanced against these strengths, limitations need to be acknowledged. First, our self-report data have the inherent problem of common method variance. Second, we relied on the convenience sample method as recruitment strategy, and our sample comprised athletes of a single nationality (i.e., Dutch), which limits the generalizability of the findings to more diverse cultural or national contexts. Further research is needed to test whether the current results can be replicated and extended across nations and cultures, within samples recruited through other sampling strategies, and in samples comprising other mixes of individual and team sports. Third, we drew on a major dichotomy in sports: individual vs. team sports. Categorizing sports as individual or team sports, however, is not as straightforward as it appears at first glance [cf., (15)]. For example, tennis is typically considered an individual sport, but tennis players also tend to play doubles in dyadic tennis teams. In sports such as baseball and cricket, the team scores determine who won or lost, but these sports may also be considered individual sports played in a team context. Hence, a finer understanding of basic psychological need satisfaction in the sport context can be obtained by making comparisons between sport events characterized by different types of interdependence [cf., (54)]: (1) no interdependence (individual races, single performance), (2) outcome interdependence only (co-acting, e.g., team competition in gymnastics), (3) sequential task interdependence and outcome interdependence (e.g., relays in athletics and swimming), and (4) reciprocal task interdependence and outcome interdependence (interactive team sports such as volleyball and basketball).

A fourth limitation of the current study is its cross-sectional design, which precludes conclusions about causality or the underlying processes driving the observed associations. Future research should explore longitudinal patterns in the relationship between sport type and basic psychological need satisfaction. For instance, individuals who experience high satisfaction of a specific psychological need may be more likely to select sports they perceive as offering greater opportunities to further fulfill that particular need. Alternatively, particular sports may inherently support certain psychological needs to a greater extent, thereby leading to higher levels of need satisfaction among athletes engaged in those sports. Furthermore, future research should explore whether, and how, individual and team sport contexts differ in the extent to which they support autonomy, relatedness, and competence needs. Another question that may be addressed in future research is whether the tendency to make self-serving attributions is indeed stronger among team sport athletes than among individual sport athletes. And if so, does that favor team sport athletes’ competence satisfaction particularly in the short term? That is, in the long term, their development and growth might suffer as performance correction strategies are less likely to be implemented (20, 56, 57).

Fifth, in our studies, we neglected individual differences in need strength. Are these nonexistent or irrelevant, as stated by SDT? From another theoretical perspective [i.e., MDT; (47–49)], individual sports may be a better fit for individuals high in need for autonomy whereas team sports may be more suited to individuals higher in needs for relatedness and competence, which may be tested in future studies. Furthermore, a limitation of the current research is that we focused exclusively on need fulfillment, and accordingly, on autonomous motivation as predictor of well-being indices [e.g., (9, 14, 44)]. SDT research suggests that need frustration is especially predictive of ill-being above and beyond low need satisfaction (12, 58). Future research may explore the added predictive value of need frustration in individual vs. team sport contexts.

In conclusion, our findings are largely in line with SDT, suggesting that the satisfaction of each basic psychological need is independently important for athletes’ autonomous motivation in both individual and team sports [e.g., (9)]. No significant differences were found in levels of autonomous motivation between individual and team sport athletes; however, the underlying sources of need satisfaction differed by sport modality. Specifically, athletes in individual sports reported higher autonomy satisfaction, whereas those in team sports reported higher relatedness satisfaction and, in Study 2 only, higher competence satisfaction. These findings offer valuable insights into athletes’ psychological need satisfaction profiles and can guide practitioners in tailoring their support to effectively nurture specific psychological needs based on sport type.

## Data Availability

The raw data supporting the conclusions of this article will be made available by the authors, without undue reservation.
